# Genome Sequence of *Anaerobacillus macyae* JMM-4^T^ (DSM 16346), the First Genomic Information of the Newly Established Genus *Anaerobacillus*

**DOI:** 10.1128/genomeA.00922-15

**Published:** 2015-08-13

**Authors:** Jie-ping Wang, Bo Liu, Guo-hong Liu, Ci-bin Ge, Qian-qian Chen, Yu-jing Zhu, Zheng Chen

**Affiliations:** Agricultural Bio-Resources Research Institute, Fujian Academy of Agricultural Sciences, Fuzhou, Fujian, China

## Abstract

*Anaerobacillus macyae* JMM-4^T^ (DSM 16346) is a Gram-positive, spore-forming, strictly anaerobic, and arsenate-respiring bacterium. Here, we report the 4.26-Mb genome sequence of *A. macyae* JMM-4^T^, which is the first genome information of the newly established genus *Anaerobacillus*.

## GENOME ANNOUNCEMENT

The *Bacillus*-like strain JMM-4^T^ was isolated from an arsenic-contaminated environment in Bendigo, Victoria, Australia (1), and was formerly identified as one member of the genus *Bacillus*, for which the name *Bacillus macyae* sp. nov. was proposed (2). In 2009, the new genus *Anaerobacillus* within the family *Bacillaceae* was established (3). Meanwhile, *B. macyae* along with *Bacillus alkalidiazotrophicus* (4) and *Bacillus arseniciselenatis* (5) were transferred to the new genus *Anaerobacillus* as *A. macyae* comb. nov., *A. alkalidiazotrophicus* comb. nov., and *A. arseniciselenatis* comb. nov., respectively (3).

Counting *A. alkalilacustre*, four species were assigned taxonomically to this genus. The mainly common features shared by all the four species of the genus *Anaerobacillus* are strictly anaerobic or aerotolerant, halotolerant or moderately halophilic, obligate or moderately alkaliphilic, chemo-organotrophic, and diazotrophic (3). More importantly, these species are capable of anaerobic respiration with arsenate as a terminal electron acceptor, using a variety of substrates as the electron donors (2, 3). Given the taxonomic history, physiological properties, and no available genomic information of *A. macyae*, its type strain F12^T^ was selected as one of the research objects in our “genome sequencing project for genomic taxonomy and phylogenomics of *Bacillus*-like bacteria.” Here, we present the high-quality draft genome sequence of *A. macyae* JMM-4^T^ (DSM 16346).

The genome sequencing of *A. macyae* JMM-4^T^ (DSM 16346) was performed via the Illumina Hiseq 2500 system. A PCR free-DNA library with insert sizes of 500 bp was constructed and sequenced using the 2 × 150-bp paired-end sequencing strategy. After filtering of the 567.42 Mb of raw data, 567.25 Mb of clean data were obtained, providing more than 100-fold coverage. The reads were assembled via the SOAPdenovo software version 1.05 (6), using a key parameter K setting at 77. Through the data assembly, 16 scaffolds with a total of 4,261,692 bp were obtained, and the scaffold *N*_50_ was 1,507,096 bp. The average length of the scaffolds was 266,355 bp, and the longest and shortest scaffolds were 2,061,986 bp and 1,383 bp, respectively. Moreover, 91.78% clean reads were aligned back to the genome, which covered 99.99% of the sequence.

Genome annotation was completed using the NCBI Prokaryotic Genomes Automatic Annotation Pipeline (PGAAP). The tRNA and rRNA genes were identified by tRNAscan-SE (7) and RNAmmer (8), respectively. A total of 4,208 genes were predicted, including 3,973 coding sequences (CDS), 145 pseudogenes, 81 tRNAs, and 8 rRNA genes. The average DNA G+C content was 39.85%, with a slight difference from the value of 37 mol% acquired by HPLC determination (2).

The genome sequence of *A. macyae* JMM-4^T^ (DSM 16346) provides useful information for genomic taxonomy and phylogenomics of *Bacillus*-like bacteria. Moreover, the genomic information will promote research on the biological significance and application in environmental biotechnology of the arsenate-respiring activity of the *Anaerobacillus* species.

### Nucleotide sequence accession numbers.

This whole-genome shotgun project has been deposited at DDBJ/EMBL/GenBank under the accession number LELK00000000. The version described in this paper is version LELK01000000.
